# A scoping review and evidence gap analysis of clinical AI fairness

**DOI:** 10.1038/s41746-025-01667-2

**Published:** 2025-06-14

**Authors:** Mingxuan Liu, Yilin Ning, Salinelat Teixayavong, Xiaoxuan Liu, Mayli Mertens, Yuqing Shang, Xin Li, Di Miao, Jingchi Liao, Jie Xu, Daniel Shu Wei Ting, Lionel Tim-Ee Cheng, Jasmine Chiat Ling Ong, Zhen Ling Teo, Ting Fang Tan, Narrendar RaviChandran, Fei Wang, Leo Anthony Celi, Marcus Eng Hock Ong, Nan Liu

**Affiliations:** 1 Centre for Quantitative Medicine, Duke-NUS Medical School, Singapore, Singapore; 2 College of Medical and Dental Sciences, University of Birmingham, Birmingham, UK; 3 University Hospitals Birmingham NHS Foundation Trust, Birmingham, UK; 4 National Institute for Health and Care Research Birmingham Biomedical Research Centre, University of Birmingham, Birmingham, UK; 5 Antwerp Center on Responsible AI, Department of Philosophy, University of Antwerp, Antwerp, Belgium; 6 Center for Medical Science and Technology Studies, Copenhagen University, Copenhagen, Denmark; 7 Department of Health Outcomes and Biomedical Informatics, University of Florida, Gainesville, FL USA; 8 Singapore Eye Research Institute, Singapore National Eye Centre, Singapore, Singapore; 9 Department of Diagnostic Radiology, Singapore General Hospital, Singapore, Singapore; 10 Department of Pharmacy, Singapore General Hospital, Singapore, Singapore; 11 Department of Population Health Sciences, Weill Cornell Medicine, New York, NY USA; 12 Laboratory for Computational Physiology, Massachusetts Institute of Technology, Cambridge, MA USA; 13 Division of Pulmonary, Critical Care and Sleep Medicine, Beth Israel Deaconess Medical Center, Boston, MA USA; 14 Department of Biostatistics, Harvard T.H. Chan School of Public Health, Boston, MA USA; 15 Programme in Health Services and Systems Research, Duke-NUS Medical School, Singapore, Singapore; 16 Department of Emergency Medicine, Singapore General Hospital, Singapore, Singapore; 17 NUS Artificial Intelligence Institute, National University of Singapore, Singapore, Singapore

**Keywords:** Health care, Medical research

## Abstract

The ethical integration of artificial intelligence (AI) in healthcare necessitates addressing fairness. AI fairness involves mitigating biases in AI and leveraging AI to promote equity. Despite advancements, significant disconnects persist between technical solutions and clinical applications. Through evidence gap analysis, this review systematically pinpoints the gaps at the intersection of healthcare contexts—including medical fields, healthcare datasets, and bias-relevant attributes (e.g., gender/sex)—and AI fairness techniques for bias detection, evaluation, and mitigation. We highlight the scarcity of AI fairness research in medical domains, the narrow focus on bias-relevant attributes, the dominance of group fairness centering on model performance equality, and the limited integration of clinician-in-the-loop to improve AI fairness. To bridge the gaps, we propose actionable strategies for future research to accelerate the development of AI fairness in healthcare, ultimately advancing equitable healthcare delivery.

## Main

Ensuring fairness in artificial intelligence (AI) in high-stakes fields like healthcare has become a paramount ethical concern, garnering considerable attention in recent years^1–5^. In-depth studies have shed light on the extensive adoption of AI techniques across diverse medical fields, while also underscoring fairness as a critical concern in ensuring ethical AI integration in healthcare^6–11^. AI bias, a systematic partiality or inclination or predisposition for or against individuals or groups with certain attributes such as gender/sex and race/ethnicity, can occur at any stage throughout the AI development lifecycle and may disadvantage certain groups or individuals over others (Box 1)^12–14^. Health equity—the principle of providing equal opportunity for all human beings to attain their full health potential regardless of societal barriers^15^, is jeopardized by such biases. With a lack of commitment to fairness, AI techniques can potentially exacerbate, rather than diminish, health inequalities^16,17^.

The context-specific nature of fairness (i.e., opposed to bias) in healthcare adds complexity to developing fair algorithm solutions^17,18^. AI fairness techniques—encompassing the evaluation and mitigation of AI biases while leveraging AI tools to advance fairness and health equity—differs markedly across medical fields, precluding a one-size-fits-all solution. First, bias-relevant attributes (some referred to as relevant attributes^19^), extending beyond frequently recorded sensitive variables such as age, gender/sex, and race/ethnicity, vary across medical fields and their bias-inducing mechanisms. For instance, in dermatology, AI bias typically arises from the underrepresentation of dark skin tones in training data, leading to lower diagnostic accuracy and higher misdiagnosis risks for darker-skinned patients^20^. In gastroenterology and hepatology, particularly liver transplantation, AI bias can arise when sex differences in clinical predictors are overlooked^7,21^. For example, the Model for End-Stage Liver Disease (MELD) algorithm, which relies on creatinine, tends to underestimate renal dysfunction in women, lowering their likelihood of receiving a liver transplant^21^. This bias occurs because women typically have lower creatinine volumes than men, but the MELD algorithm applies the same reference standard to both sexes^21^.

Additionally, different medical fields rely on distinct data types, each posing unique fairness challenges. For instance, radiology and pathology predominantly deal with imaging data, which is presumably more objective for decision-making but can still contain hidden biases^22^. In contrast, mental health often depends on self-reported outcomes and behavioral data, which are more subjective and susceptible to human cognitive bias from both patients and healthcare providers^8,23^. When quantifying fairness, the perspectives also vary, reflecting the complex interplay between bias-relevant attributes and the outcomes of interest. In radiology, fairness emphasizes equality in model performance (e.g., in terms of equal false positive rates; corresponding to “performance-based” metric in Box 1) across demographic subgroups (e.g., between male and female), despite varied biological detection challenges^24,25^. In liver transplantation, fairness is guided by medical urgency over socioeconomic status to guarantee equitable treatment access and equality decision-making (e.g., in terms of equal decision rates; corresponding to “parity-based” metric in Box 1) across all subgroups^26^.

While extensive works have narratively explored different aspects of AI fairness in healthcare^27–31^, a comprehensive understanding of its current status in advancing health equity remains absent. A significant disconnect persists between AI fairness techniques and healthcare calls, leading to numerous discussions but limited progress in clinical AI fairness^17,32^. This review aims to systematically pinpoint the deficiencies at the intersection of healthcare contexts (encompassing various medical fields, bias-relevant attributes and datasets) and AI fairness techniques (including bias detection, evaluation, and mitigation) from a quantitative perspective. By employing evidence maps, we highlight critical gaps, such as the scarcity of AI fairness research in many medical domains, the narrow focus on bias-relevant attributes, the dominance of group fairness approaches centering on model performance equality, and the limited integration of clinician-in-the-loop to improve AI fairness. We also propose actionable strategies to accelerate the development and adoption of clinical AI fairness, bridging the gap to promote fair and equitable healthcare delivery.

Box 1 Terminology box

**Table:** 

Terminology		Description	Example
**Concept terminology**			
Health equity		A fundamental human right, achieved when everyone attains their full potential for health and well-being	Two people born with different health needs ultimately obtain the same health and well-being
Bias		A systematic partiality or an inclination or predisposition for or against individuals or groups with certain attributes, resulting in disadvantaging certain groups or individuals over others	Perception that Black individuals have a higher pain tolerance, leading to the systematic undertreatment of their pain
Fairness		Fairness is defined as opposed to bias to promote human equity	Two patients with similar health conditions receive treatments to obtain similar outcomes, despite differences in attributes such as race/ethnicity
AI bias		Bias occurring at any stage of the AI development lifecycle, from data collection to algorithm implementation	A pulse oximetry algorithm systematically exhibiting lower accuracy for individuals with darker skin tones
AI fairness		Absence of AI bias. AI fairness techniques refer to efforts to identify, evaluate and mitigate AI biases while leveraging AI tools to advance fairness and health equity	Ensuring an algorithm takes skin tone into consideration when screening for skin cancer while not having skin tone weigh inappropriately in the access or quality of care
Bias-relevant attributes		Attributes that can directly or indirectly link to AI biases in decision-making and access to resource	Skin tone affecting dermatological diagnosis accuracy
**Fairness methods**			
Pre-process		To remove bias in the model development data	Balancing data distribution between subgroups (resampling); aligning data distribution with population demographics (reweighting)
In-process		To build a model with properties which are intended to optimize fairness and/or reduce bias, with original data that may or may not have bias	Filtering bias-related information in representation learning; adding regularization to objective function; subgroup-wise modeling
Post-process		To modify the existed model according to different subpopulations before and during the implementation	Optimizing thresholds for subgroups
**Fairness metrics**			
Group fairness	Parity-based	Focusing on predicted positive values	Demographic (statistical) parity, disparate impact
	Performance-based	1) Addressing the equality of performance metrics (e.g., accuracy, sensitivity [TPR], specificity [TNR], etc.) among different subgroups 2) Focusing on the equality of calibration between average predicted probability and fractions of positive values	Equal opportunity, equalized odds, other metrics computed by differences or ratios of machine learning metrics among subgroups
	Rank-based	Focusing on the relative ranking of scores among outcome classes (e.g., mortality and survival), expected to be independent on group identity regarding bias-relevant attributes	Disparity in bipartite-ranking metrics
	Remove-based	Focusing on the removal of bias-related attributes or confounders	Under blindness which to directly remove bias-relevant attributes, Mutual information measuring the removal of bias-relevant attributes
Individual fairness	Similarity-based	Emphasizing similar results from similar individuals	Fairness with awareness which utilizes mathematically defined similarity
	Counterfactual-based	Emphasizing unchanged results after changing the bias-related attributes of individuals	Counterfactual fairness
Distribution fairness	Variance-based	Emphasizing the equality of quantities received by participants via minimizing variation	Variance or standardized deviation of the quantities (e.g., accuracy, loss, etc.) among participants
	Reward-based	Emphasizing alignments between the quantities received by participants and their inputs and efforts	Reward based on the size of dataset

## Results

**Fig. 1:**
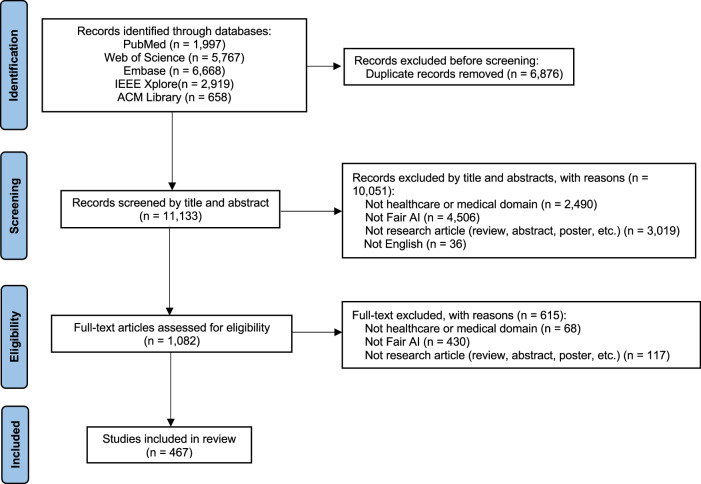
PRISMA-ScR flow diagram.

We conducted a systematic scoping review to analyze the current landscape of AI fairness research in healthcare. Our search of five databases (MEDLINE, Web of Science, Embase, IEEE Xplore, ACM library) yielded 11,133 unique papers, of which 467 were included for analysis. Figure 1 illustrates the selection procedure in detail. Our evidence gap analysis uncovered critical deficiencies in clinical AI fairness, specifically in the applications of AI fairness across various medical specialties, the bias-relevant attributes considered, and the clinical datasets employed, which shape the landscape of AI fairness through the clinical lens. Upon this, we pinpointed gaps in the methodologies used to quantitatively identify, evaluate, and mitigate biases from the technical perspective.

### Medical fields and healthcare data: an overview

**Fig. 2:**
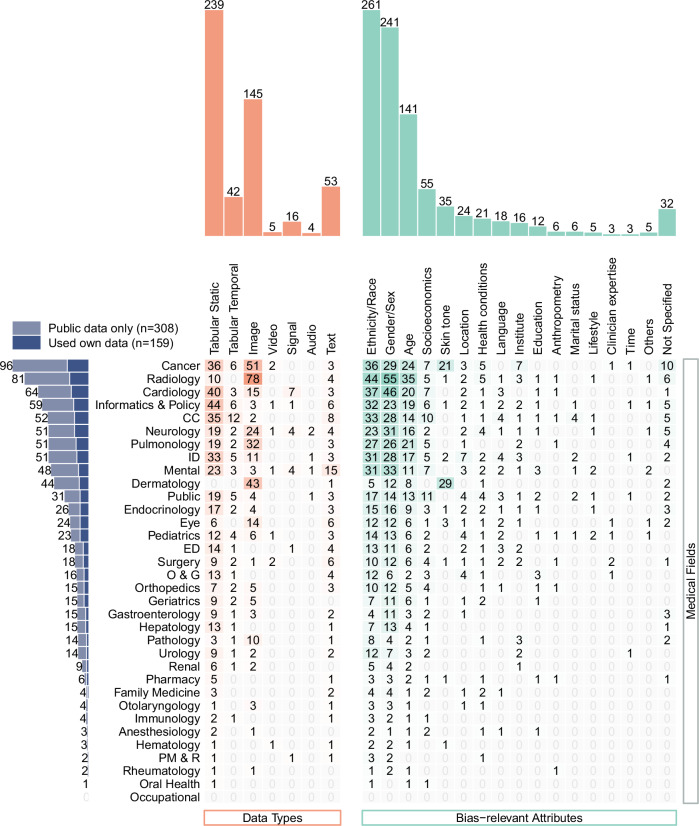
The evidence gap analysis of AI fairness methodology developments and applications in cross-tabulation between medical fields and data types, bias-relevant attributes, and public dataset utilization. Each unit (“1”) represents a single paper, where one paper may use multiple datasets, and each dataset can encompass various specialties, data types, and bias-relevant attributes. Papers were classified as “Public data only” if all datasets used were public; otherwise, they were classified as “Used own data“. CC Critical Care, ED Emergency Department, ID Infectious Diseases.

Figure 2 summarizes the distribution of AI fairness studies across various medical fields. The category of health informatics and policy encompasses studies focused on health policy as well as those not fit into a specific medical field, such as predicting length of stay in uncertain settings. Figure 2 also classifies studies by data types, bias-relevant attributes, and the use of publicly accessible datasets. All reviewed studies were based on retrospective data. AI fairness research was limited in several medical fields (i.e., appeared in less than five papers), including otolaryngology, family medicine, immunology, anesthesiology, hematology, physical medicine and rehabilitation, rheumatology, oral health, and occupational health.

Regarding data types, tabular static data emerged as the most frequently used data type (n = 239, 51.2%), prevalent in various medical fields. Image data, ranking second (n = 145, 31.0%), was primarily used in specialized areas such as cancer, radiology, and dermatology. Tabular temporal data was occasionally used in research across 22 medical fields. Other data types, such as video, signal, audio, and text, were used less frequently in the studies reviewed. Among all medical fields, the analysis of AI fairness in mental health exhibited the broadest diversity in data types, utilizing all seven categories, predominantly tabular static and text data. Conversely, AI fairness studies in dermatology mainly concentrated on image data.

Among the 467 included papers, most papers (n = 308, 66.0%) only analyzed publicly available datasets (see Fig. 2). We summarized a total of 241 publicly accessible datasets, grouped by data type and ordered by frequency. Most of these public datasets were only used in less than five papers in this review (n = 226, see Supplementary Table 1). Table 1 lists the datasets that were employed more frequently, covering four data types: tabular static, tabular temporal, image, and text data.

**Table 1:** Popular public datasets for AI fairness investigation

Data type	Public dataset	Medical fields^a^	Bias-relevant attributes	Number of papers
Tabular Static	MIMIC-III, MIMIC-IV	CC, ED, Geriatrics, ID, Neurology, Orthopedics, Pharmacy, Pulmonary, Renal, Surgery	Age, Education, Ethnicity/Race, Gender/Sex, Language, Lifestyle, Marital status, Socioeconomics, Not Specified	24
	MEPS	Informatics & Policy, Oral Health	Age, Ethnicity/Race, Gender/Sex, Socioeconomics, Not Specified	13
	Heritage Health	Informatics & Policy	Age, Gender/Sex, Not Specified	11
	UCI Heart Disease dataset	Cardiology	Age, Gender/Sex	8
	SEER	Cancer, Gastroenterology, Hepatology, Pathology, Pulmonary, Urology	Age, Ethnicity/Race, Gender/Sex, Socioeconomics	7
	SUPPORT	Cancer, Cardiology, CC, Gastroenterology, Hepatology, ID, Neurology, Pulmonary	Age, Ethnicity/Race, Gender/Sex, Not Specified	6
	UCI diabetes dataset	Cardiology, Endocrinology	Age, Education, Ethnicity/Race, Gender/Sex, Not Specified	6
	eICU	CC, Geriatrics	Age, Ethnicity/Race, Gender/Sex, Institute, Language	5
Tabular Temporal	MIMIC-III, MIMIC-IV	CC, ID	Age, Ethnicity/Race, Gender/Sex, Language, Marital status, Socioeconomics	9
Image	ISIC (HAM10000, BCN20000, etc.)	Cancer, Dermatology, Radiology	Age, Ethnicity/Race, Gender/Sex, Health conditions, Skin tone, Not Specified	20
	CheXpert	Cardiology, Dermatology, Pulmonary, Radiology	Age, Ethnicity/Race, Gender/Sex, Skin tone	18
	Fitzpatrick17k	Cancer, Dermatology	Age, Gender/Sex, Health conditions, Skin tone	13
	MIMIC-CXR	Cardiology, Dermatology, Pulmonary, Radiology	Age, Ethnicity/Race, Gender/Sex, Skin tone, Socioeconomics	13
	Chest-Xray8, Chest-Xray14	Cardiology, ID, Pulmonary, Radiology	Age, Ethnicity/Race, Gender/Sex	12
	ADNI	Geriatrics, Neurology, Radiology	Age, Gender/Sex	5
	EyePACS	Endocrinology, Eye	Age, Ethnicity/Race, Gender/Sex, Health conditions, Skin tone, Not Specified	5
Text	MIMIC-III, MIMIC-IV	CC, Mental	Ethnicity/Race, Gender/Sex, Not Specified	6

^a^refers to those medical fields where the databases were applied as observed in this review, rather than how these databases are defined.

The complete list of public datasets used for AI fairness in this review can be found in Supplementary Table 1.

Abbreviations:

*MIMIC-III / MIMIC-IV* Medical Information Mart for Intensive Care III / IV clinical databases,

*MEPS* Medical Expenditure Panel Survey,

*Heritage* Health Heritage Health Prize dataset,

*SEER* Surveillance, Epidemiology, and End Results Program,

*SUPPORT* Study to Understand Prognoses and Preferences for Outcomes and Risks of Treatments,

*eICU* eICU collaborative research database,

*ISIC* International Skin Imaging Collaboration challenge datasets,

*CheXpert* Chest eXpert dataset (Stanford University),

*ChestX-ray8 / ChestX-ray14* National Institutes of Health (NIH) Chest X-ray Dataset with 8 or 14 labeled pathologies,

*ADNI* Alzheimer’s Disease Neuroimaging Initiative,

*EyePACS* EyePACS diabetic retinopathy dataset,

*CC* Critical Care,

*ED* Emergency Department,

*ID* Infectious Diseases.

### Bias-relevant attributes: patterns and skewness

The most prevalent bias-relevant attributes investigated in AI fairness studies were ethnicity/race (*n* = 261, 55.9%) and gender/sex (*n* = 241, 51.6%), followed by age (*n* = 141, 30.2%). These concept pairs – gender and sex, ethnicity and race – often lacked precision in reporting by the authors, so they were collectively summarized. Other than these three most examined bias-relevant attributes, socioeconomic status was featured prominently in studies, followed by skin tone, location, health condition, language, institute (e.g., medical center, hospital), and education. Among these, skin tone was particularly popular for skin cancer studies, location was prevalent for infectious disease, and institute was frequently concerned in cancer studies.

Additional attributes like anthropometry (weight and height), marital status, lifestyle, the experience level of clinicians, time dynamics, etc., were considered for AI fairness in at least three papers each. The category of “Others” included five papers that dealt with bias-relevant attributes including religion, sexual orientation, name, intelligence quotient, eye color, each only appearing once in this review. Thirty-two papers did not specify bias-relevant attributes. Most focused on fairness notions not requiring specific bias-relevant attributes, for example, Liu et al^33^. calculated individual similarities without bias-relevant attributes. Others were purely methodological papers that symbolized bias-relevant attributes^34^.

### Bias identification

A total of 267 studies (57.2%) positioned bias identification as a precursor to bias mitigation methods. These studies commonly leveraged literature evidence^35,36^, exploratory data analysis^37,38^, and method comparisons to detect biases^39,40^. Data bias was often quantified by class imbalances and underrepresentation of minorities, which may intrinsically lead the model constructed to perform badly for these populations^37,41^. Algorithmic bias was mainly detected using fairness metrics (see Box 1 and subsection “Bias evaluation metrics”) through comparison across algorithms. Despite some studies attempting to clarify the underlying causes and mechanisms of biases (e.g., Park et al.^38^), most of them remained elusive largely due to insufficient contextual analysis.

In contrast, 200 papers (42.8%) focused solely on bias identification without providing bias mitigation solutions. These studies typically employed regression models to identify biases in the data by examining the association between bias-relevant attributes and the outcomes of interest^42,43^. Algorithmic bias was frequently identified by comparing model performance between subgroups, with performance variations as evidence of bias^44,45^.

Among the included papers, 62 (13.3%) explicitly mentioned the inclusion or exclusion of bias-relevant attributes as predictors. Bias identification approaches varied by algorithm type: machine learning models (e.g., random forests, support vector machines [SVM], linear models) more frequently clarified whether bias-relevant attributes served as predictors^46,47^, while deep learning (DL) models more often emphasized whether the model could directly predict such variables^48,49^. Generative AI (GenAI), particularly large language models (LLMs), primarily focused on prompt design in medical contexts and its influence on demographic representation in outputs^50,51^.

### Bias evaluation metrics

**Fig. 3:**
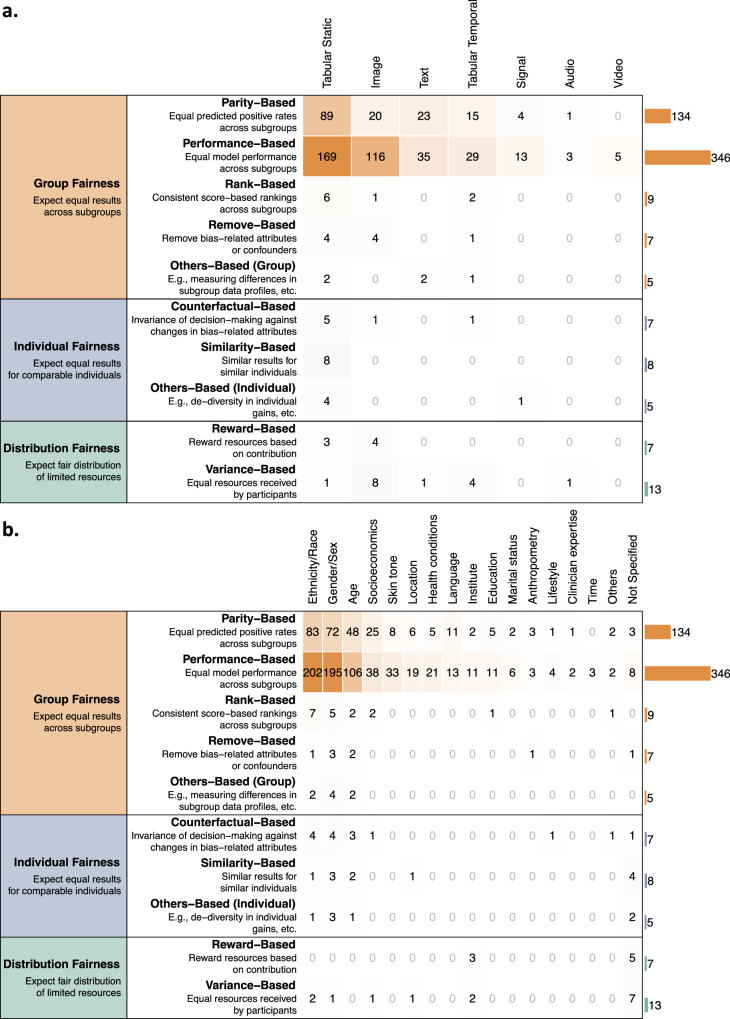
The evidence gap analysis of fairness metrics across fairness notions and data characteristics. The evidence gap analysis of fairness metrics, cross-tabulated by high-level fairness notions (group fairness, individual fairness and distribution fairness) against **a** data types and **b** bias-relevant attributes. Each unit (“1”) represents a single paper. One paper may involve multiple fairness notions and metrics.

Bias evaluation is crucial to detecting algorithmic bias and to assessing the efficacy of bias mitigation methods if applied (details explained in Box 1). As shown in Fig. 3, group fairness was the most common fairness notion (*n* = 435, 93.1%), while individual fairness (*n* = 20, 4.3%) and distribution fairness (*n* = 18, 3.9%) were mentioned less often (see Box 1 and Fig. 3 for more details). We did not observe obvious differences across algorithm types (Supplementary fig. 1).

Group fairness (*n* = 435) was dominated by performance-based (*n* = 346, 79.5%) and parity-based (*n* = 134, 30.8%) metrics, such as equal opportunity^52^, equalized odds^52^, and statistical (demographic) parity^53^. These two types of metrics covered nearly all data types and bias-relevant attributes reviewed (Fig. 3) and were jointly utilized in 63 (14.5%) papers^54,55^. Image- or video-based studies predominantly relied on performance-based metrics (Fig. 3a). Performance-based metrics typically gauge disparities in model performance, such as accuracy, true positive rate, etc. However, calibration performance—reflecting the degree to which the predicted probabilities match actual outcomes, was infrequently considered among the included studies (*n* = 14, 3.2%)^46,56^. Rank-based metrics (*n* = 9, 2.1%), used for continuous predictions to ensure consistent outcome distributions across subgroups, were primarily employed for survival predictions in cancer and critical care studies using tabular static data^47,56,57^. Remove-based metrics (*n* = 7, 1.6%), which focus on measuring the removal of confounders or disentanglement of bias-related information, were mainly applied to tabular static and image data^49,58^.

For studies on individual fairness (*n* = 20), tabular static data predominated, with bias-relevant attributes primarily including ethnicity/race, gender/sex, age, or unspecified, as shown in Fig. 3. Similarity-based metrics (*n* = 8, 40.0%) were the most commonly used for individual fairness, expecting comparable model outputs of similar individuals^59–61^ and often without relying on specific bias-relevant attributes^60,61^ (Fig. 3b). Counterfactual-based metrics (*n* = 7, 35.0%) emphasized prediction invariance to artificial changes in attributes, e.g., change of a female identity into a male, and typically leveraged causal inference for fairness analysis^62,63^.

For studies on distribution fairness (*n* = 18), the allocation of healthcare resources also did not necessarily specify bias-relevant attributes, dominated by image data (Fig. 3). Participants in distribution fairness schemes could be both individuals and groups that are usually geographically apart (for example, patients from different hospitals). Among these studies, two types of objectives prevailed: to distribute equally among the participants, where variance-based metrics were applied^64,65^ (*n* = 13, 72.2%), or to distribute appropriately based on the efforts and inputs of participants, where the reward-based metrics were applied^66,67^ (*n* = 7, 38.9%).

### Bias mitigation

**Fig. 4:**
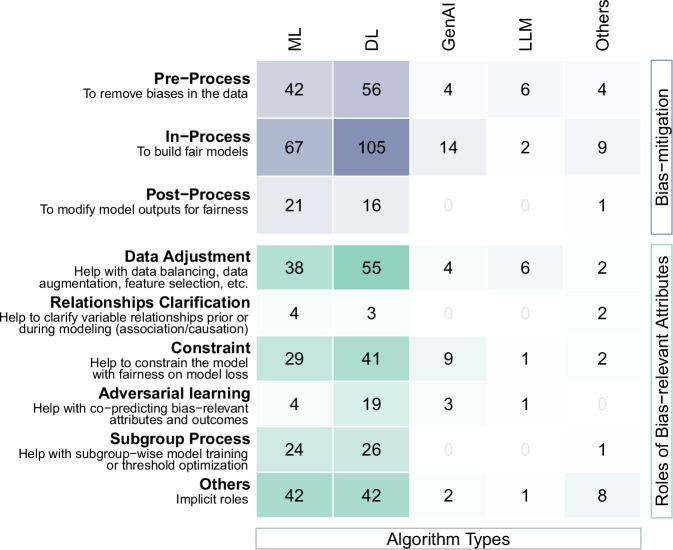
The evidence gap analysis of fairness methods and roles of bias-relevant attributes in cross-tabulation with algorithm types. Each unit (“1”) represents a single paper. One study might contain several types of bias mitigation methods, and the bias-relevant attributes could have different roles in bias mitigation.

Out of the 267 papers that attempted to mitigate bias, in-process methods were the most common (*n* = 176, 65.9%), followed by pre-process (*n* = 96, 36.0%) and post-process (*n* = 31, 11.6%) methods. This indicates constructing inherently fair models (in-process; see Box 1 for detailed definition) was favored over adjusting data beforehand (pre-process) or correcting model outputs afterward (post-process). However, studies involving LLMs favored pre-process bias mitigation methods (Fig. 4). Some papers adopted multiple types of methods (*n* = 30, 11.2%), either by comparing existing methods^38,68^ (*n* = 19) or developing fairness methods to mitigate bias across multiple stages^69,70^ (*n* = 11).

Pre-process methods (n = 96) commonly adjust data distribution via resampling (such as under-sampling^37^ and fairness-enhanced sampling^71^), reweighing^38,72^, and augmentation^73,74^ strategies to balance data between subgroups or align it with population demographics (*n* = 82, 85.4%). Some papers investigated data adaptation (*n* = 7), utilizing constructed models to transfer fairness to local data^75,76^. Ten papers refined feature selection to improve model fairness, such as incorporating social determinants of health^77,78^. In-process methods (*n* = 176) frequently constrained models with fairness metrics^38,72,79^ (*n* = 73, 41.5%), applied representation learning particularly via adversarial learning^80,81^ (*n* = 25, 14.2%) to address fairness by filtering bias-related information when modeling, or subgroup-wisely constructed models (*n* = 22, 12.5%). Post-process methods (*n* = 31) generally optimized prediction thresholds for subgroups (*n* = 19, 61.3%)^81,82^. However, addressing fairness during model implementation, or constructing post-modeling clinical pathways to mitigate bias were rare in this review^83^.

Bias mitigation varied by AI algorithms (Fig. 4). Discriminative DL models dominated (*n* = 155, 58.1% over 267 bias mitigation studies) regardless of bias mitigation methods. Particularly, adversarial learning was frequently leveraged to co-train predictions of bias-relevant attributes and outcomes for fair DL models. Studies involving GenAI and LLM seldom clarified the relationships (association, causation, etc.) between bias-relevant attributes and other variables to enhance fairness. Additionally, none of these studies conducted subgroup-wise modeling or post-processing for fairness.

### Emerging topics for clinical AI fairness

Forty-one papers used explainable artificial intelligence (XAI) to enhance fairness assessments in clinical AI. The majority (*n* = 32) utilized XAI to explore bias pathways, such as identifying bias-relevant attributes as significant contributors to predictions^84,85^, uncovering other factors driving model bias for further investigations^42,86^, and analyzing differences in variable importance across subgroups^69,87^. Common XAI methods, such as SHapley Additive exPlanations (SHAP)^88^ and Gradient-weighted Class Activation Mapping (GradCAM)^89^, were frequently utilized, while some studies introduced model-specific measures to contextualize bias sources and inform fairness evaluations^90–92^. To demonstrate the impact of bias mitigation, XAI was also applied to analyze changes in variable importance^93,94^, guide fairer model development by excluding bias-inducing parts (e.g., masking specific model parameters)^95^, and improve model fairness by aligning model explanations with expert-derived explanations^96^.

In this review, clinician-in-the-loop (defined as the active involvement of healthcare professionals in the development, implementation, and utilization of AI systems^17^) played a pivotal role in advancing AI fairness, with clinicians’ involvement documented in 33 studies. These contributions can be classified into three primary roles: evaluation and validation (*n* = 16), where clinicians assessed model outputs for accuracy and fairness^97,98^; decision support experiments (*n* = 12), where clinicians participated in assessing clinical impact of AI models and investigating the modes of human-AI interaction^99–101^; and supervision (*n* = 5), where clinicians provided direct feedback to refine AI systems to promote fairness^96,102^.

Twenty-one studies employed federated learning (FL)—a framework that allows for cross-site collaboration while preserving data privacy^66,67^. As an in-process bias mitigation approach, FL with fair aggregation mechanisms was primarily used to promote distribution fairness, addressing the fairness in quantity received among participants (*n* = 13). These studies employed discriminative DL^66,67^ or GenAI^103^. In addition, FL was also used to address group fairness (*n* = 8), often integrated with other bias mitigation techniques such as model constraints^65,104^ and adversarial learning^105,106^. These studies typically focused on race/ethnicity and gender/sex biases at each site, using performance-based metrics.

## Discussion

Despite growing attention to clinical AI fairness, a comprehensive picture of its status in advancing health equity is still lacking. Our scoping review of 467 studies revealed critical gaps: limited applications of AI fairness in most medical fields, narrow consideration of bias-relevant attributes, the dominance of group fairness, and minimal clinical-in-the-loop. These findings underscore a misalignment between the development of AI fairness techniques and the context of clinical applications. Bridging this gap will require stronger interdisciplinary collaboration between technological and clinical experts. To guide future efforts, we outline fundamental challenges in clinical AI fairness, propose actionable strategies for its advancements, and suggest future directions for both AI developers and clinical researchers, as summarized in Table 2 and described below.

**Table 2:** Challenges of clinical AI fairness and how they may be overcome with future development

Challenges	Current status	Strategies
Advancing AI fairness research across medical fields	Insufficient emphasis of AI fairness across various medical domains	− Identify and summarize well-established databases suitable for AI fairness investigation in each medical field − Collect and curate medical field-specific datasets − Promote the sharing of medical data with strong privacy protection mechanisms
Beyond sensitive variables	Inadequate consideration of diverse factors that introduce biases in healthcare data.	− Actively identify and discuss factors that may induce biases − Ensure precise terminology when describing bias-relevant attributes
Addressing individual fairness	Limited focus on fairness at the individual level.	− Consider more individual-specific factors (e.g., language literacy) − Develop bias evaluation metrics and bias mitigation methods for individual fairness.
Investigating distribution fairness	Insufficient exploration of fairness in resource allocation.	− For physical healthcare resource allocation, develop fairness-aware and decision-aware models and mechanisms. − For computational resources, address fairness through techniques like federated learning with incentive mechanisms.
Putting AI fairness into clinical contexts	Weak integration of AI fairness into practical healthcare applications.	− Develop fairness metrics for procedural fairness. − Involve clinicians in the early stages of modeling − Establish clinical guidelines for the usage of AI fairness metrics. − Develop clinical pathways for equitable health outcomes as non-technological approaches

Challenge 1: Advancing AI fairness research across medical fields. First, AI fairness research remains underexplored in many medical fields, as shown in Fig. 2, despite calls for investigation^9–11^. The lack of AI fairness studies in fields like anesthesiology and oral health indicates overlooked concerns of AI fairness, given the prevalence of AI research in these fields, as summarized in Supplementary Table 2. For example, Morch et al^11^. identified around 200 studies about AI in oral health (up to 2020), yet our review only identified one study about AI fairness in oral health. Even in fields with more AI fairness research, such as cancer and cardiology, the attention to AI fairness remains insufficient compared to the extensive AI-related studies. As AI methodologies and applications evolve, integrating fairness considerations from the outset is crucial, rather than treating them as an afterthought.

In addition, public datasets often lack standardization or are under-utilized, limiting robust fairness benchmarks for diverse clinical questions. Figure 2 shows a reliance on public databases in many fields like cancer, radiology, cardiology, health informatics and policy, and critical care. Yet, our review finds sparse use of many public datasets listed in Supplementary Table 1, often single-study applications, suggesting a deficiency in standardized fairness benchmarks concerning various clinical questions. Moreover, the infrequent use of established databases like the National Health and Nutrition Examination Survey (NHANES) for AI fairness studies indicates that the potential utility of these resources may be under-valued or that they may not be sufficient due to the absence of bias-related information^19^.

Addressing challenge 1 necessitates a multifaceted strategy. To tailor AI fairness for specific medical fields, it is crucial to summarize^107,108^ and examine^109,110^ field-specific datasets through the lens of fairness, and to collect new datasets with fairness concerns when existing data is insufficient. Concerted efforts are needed to collect data with rigorous strategies that ensure data diversity and representativeness^111^, with clinician-assisted curation to mitigate potential data bias where necessary^19,20^. Importantly, such efforts should also include improvements to medical devices to generate data with equal quality from subpopulations (e.g., when measuring blood oxygen for people with varying skin tone), and the use of less biased proxies for health-related measures (e.g., cystatin C as a gender-neutral alternative to creatinine for measuring renal function^112^). Additionally, making medical data publicly available, with attention to both access and privacy^113^, will foster thorough AI fairness research in compliance with Health Insurance Portability and Accountability Act (HIPAA) standards.

Challenge 2: Beyond sensitive variables. In general context, sensitive variables (such as age, race/ethnicity, gender/sex) are often used interchangeably with protected attributes, i.e., those protected from discrimination by law^114^. However, in clinical AI fairness, these attributes do not encompass the full spectrum of bias. Bias can arise from many variables that are not necessarily sensitive or protected. For example, as previously mentioned, parameters like creatinine may inadvertently introduce biases if reference ranges are not adequately representative of different populations^7^. Additionally, commonly mentioned sensitive variables require conceptual clarity. For example, “gender” as a social identity was often conflated with “sex” as a biological concept—a discrepancy that needs clear differentiation to enhance the validity of fairness assessments^18^.

Beyond sensitive variables, bias-relevant attributes encompass a wider range, including health status (not limited to disability status), institute, anthropometry (weight and height), experience level of clinicians, etc. Although acknowledged as bias-related, these attributes are often overlooked in clinical AI fairness. In addition, most of these attributes are patient-oriented, but characteristics of healthcare providers can also induce biases (e.g., via a patient-physician gender discordance^115^) and are seldom available for modeling. Moreover, the intersectional effects of these attributes were infrequently considered. Accurately capturing and reporting them is challenging but essential for thorough bias investigation. Furthermore, these attributes should be leveraged to actively mitigate bias rather than being used solely as proxies.

Moreover, many papers from the technical communities utilize certain datasets and bias-relevant attributes to demonstrate their bias mitigation techniques, without providing a clear justification for their handling of bias-relevant attributes. They also might not assess initial bias profiles beyond class imbalance and varying prevalence rates^111^, let alone elucidate the clinical impact of bias mitigation techniques. This necessitates further comprehension of clinical contexts and precise characterization of fairness before developing AI fairness methods.

Challenge 3: Addressing individual fairness. Although group fairness metrics (parity-based and performance-based) were frequently researched, as indicated in Fig. 3, there is a marked deficiency in individual fairness to ensure comparable individuals are treated equally. As policymakers and healthcare managers prioritize group fairness based on population ethics, individual fairness, which directly affects patients and is aligned with clinical ethics, is equally essential^17,33^. The rise in precision medicine amplifies the need for a balanced approach to AI fairness, incorporating both broad population insights and individual patient nuances to achieve equitable healthcare outcomes^116^.

The lack of investigation into individual fairness in current literature may indicate the difficulty of integrating it into clinical AI fairness. We can take an intermediate step by adopting an intersectional approach that considers multiple bias-relevant attributes simultaneously for fairness considerations to capture more accurate individual profiles. AI researchers can develop fairness metrics that measure biases at a granular level, accounting for individual variations and meticulously assessing individual similarity in clinical settings^79,117^. Subsequently, researchers can enhance individual fairness by crafting personalized treatment recommendations that accounts for individual variations in disease presentation and response to treatment while avoiding unjustified effects from bias-relevant attributes^33^. The downside is that incorporating additional personal attributes to enhance AI fairness can inadvertently increase the risk of compromising individual privacy. As it remains a subject of debate whether the distinction between individual and group fairness is merely a matter of granularity^118^, it is beneficial to explore additional potential pathways that connect group fairness with individual fairness. Moreover, algorithms should be transparent in their decision-making, ensuring individuals can comprehend the impact on their outcomes (e.g., diagnoses and treatment recommendations)^79^, which goes beyond broad group identities to recognize and respect individual patient autonomy.

Challenge 4: Investigating distribution fairness. The current research on clinical AI fairness also significantly overlooks distribution fairness, which is vital for the equitable distribution of resources across participants, whether individuals or groups. While some studies in this review addressed distribution fairness, they primarily focused on computational aspects such as data collaboration and model co-training^66,67,103^, rather than fair allocation of physical resources such as vaccines^119^. For the distribution of computational resources, these studies emphasized the equal distribution among participants (variance-based) over the balance of contribution and benefit (reward-based)^66,67^. To enhance distribution fairness, incentive-equipped FL can optimize data resource use and fairly reward participants based on model performance. Moreover, addressing bias across institutions and geographic locations is important, as variability across data sources can impact model fairness^81^.

For the distribution of physical resources, AI techniques are usually integrated with network analysis to promote fairness^119^. However, the fairness along the health supply chain remains largely overlooked, where fair AI models may integrate with optimization mechanisms^120^. Even a bias-mitigated algorithm may fail to influence biased decision-making and resource allocation in practice^121^. This highlights the need for decision-aware fairness frameworks that go beyond the algorithmic level and address the fairness challenges in the physical end.

Challenge 5: Putting AI fairness into clinical contexts. Current AI fairness techniques lack contextual ties to healthcare scenarios. Group fairness, which concentrates on “outcome fairness” (i.e., the fairness of the decision outcomes)^81,122^, makes up most evaluation metrics. In-process methods also dominate the fairness methodologies, followed by post-process methods, which usually involve “black-box” models with obscure data bias and bias mitigation pathways. This obscures “procedural fairness” (i.e., the fairness in the decision-making process)^123,124^—another perspective of fairness that is largely neglected in the current literature. Even for approaches like reinforcement learning, which addresses the interaction of model development and context, the corresponding fairness methodology typically focuses only on the final outputs^40^. This also applies to large language (or vision) models that are based on reinforcement learning and encapsulated as interfaces for healthcare applications. Moreover, the lack of clinician involvement early in model development—beyond merely evaluating model outputs—misses the opportunity to integrate clinical expertise into bias mitigation.

Clinical AI fairness is not a one-time effort, as biases can emerge at any stage and impact eventual patient outcomes^17,125^. To cultivate clinically contextualized fairness, it is essential to actively involve domain experts who are familiar with specific clinical contexts and aware of biases in the model development and evaluation process^126^. Despite some papers highlighting the role of domain experts in bias assessment and mitigation^96–98,102^, further strategies for integrating domain experts remain to be explored. This can include matching machine learning tasks with clinical problem formulation^127^, employing causal modeling with clinically precise measurements^128^ (e.g., using healthcare expenditures as a proxy for health needs is often implausible^129^), designing new paradigms for quantitative bias evaluation for unstructured model output, formulating clinical guidelines for AI fairness applications, crafting interpretable AI fairness methods to enhance dialog between all stakeholders, and so on. Moreover, in response to biased models, developing clinical pathways aimed at equitable health outcomes can provide a non-technological approach to ensure fairness in healthcare delivery. For instance, offering patients prone to underdiagnosis an additional test or an earlier evaluation by a clinician can help align model outcomes with real-world clinical decision-making^1,5^.

This review has several limitations. First, ambiguity in how some papers referenced healthcare datasets may have hindered accurate identification and counting of datasets. Second, while our classification of medical fields and demographic constructs (such as race and ethnicity) aimed to be comprehensive, our taxonomy may not fully align with all global classification systems due to international variations. Last but not least, by including only English-language papers, we may have limited the scope of our fairness discussion, potentially omitting insights on global health disparities.

The disconnect between AI fairness techniques and the urgent demands in healthcare fields is evident through the gaps in clinical applications and the development of AI fairness techniques. Joint exploration by healthcare professionals and AI researchers, preferably assisted by ethicists, is essential to expand beyond traditional sensitive variables, refine fairness quantification, and contextualize AI fairness in healthcare scenarios, thereby bridging current gaps. The pursuit of equitable and fair healthcare delivery is advancing, propelled by interdisciplinary partnerships and a shared commitment to excellence in patient care and technological innovation.

## Methods

### Search strategies

This review followed the Preferred Reporting Items for Systematic reviews and Meta-Analyses extension for Scoping Reviews (PRISMA-ScR) checklist^130^ (Supplementary Table 3). The protocol, including the research questions, is available at https://osf.io/q5k2b/. We searched five databases (MEDLINE, Web of Science, Embase, IEEE Xplore, ACM library) up to September 24, 2024. Detailed search strategies are provided in Supplementary Table 4. Seven reviewers (ML, ST, YN, YS/JL, XL, and DM) screened the articles based on the eligibility criteria. We randomly split papers into three non-overlapped groups. A pair of reviewers (ML with XL, YN with DM, and ST with YS/JS) was allocated to each group with a similar number of publications. These reviewers individually screened the respective papers. Any disagreements were resolved through discussions among the seven reviewers, under the oversight of a supervising reviewer (NL).

### Concepts of bias and fairness with AI

For this review, we defined key concepts, including health equity, bias, fairness, AI bias, AI fairness, and bias-relevant attributes, as presented in Box 1. According to the World Health Organization (WHO), health equity is a fundamental human right achieved when everyone can reach their full potential for health and well-being^15^. While bias and fairness have multifaceted meanings, we focused on the aspects related to human beings, whether individuals or groups of individuals, since they are the primary subjects of healthcare. In the general context, the American Psychological Association (APA) defines bias as a partiality, or an inclination or predisposition for or against something^12^. When it comes to the context of digital health, we followed the Standards for Data Diversity, Inclusivity, and Generalizability (STANDING Together) initiative, emphasizing bias’s systematic nature and consequences—disadvantaging certain groups or individuals over others^13,14^. Fairness is generally defined as opposed to bias, promoting health equity^17^.

Regarding AI, aligning with STANDING Together’s description, we defined AI bias as bias that can occur at any stage of the AI development lifecycle, and further specified that this includes processes from data collection to algorithm implementation. AI fairness, in turn, is considered the absence of AI bias. AI fairness techniques encompass efforts to identify, evaluate, and mitigate AI bias while leveraging AI tools to advance fairness and health equity. In quantitative practice, AI fairness techniques typically aim to quantify and enforce equality concerning specific aspects of model decision-making across individuals or groups^114^. The aspect that requires equality (e.g., positive rate, accuracy, calibration) usually depends on the medical context. These definitions guided our process for paper screening, data extraction and information synthesis.

### Exclusion criteria

Papers were excluded for any of the following reasons: the paper was not in the medical or clinical domain; the paper was not about AI fairness, meaning the study should address AI fairness with a clear description or quantification of fairness or bias; the paper was not published as a research article (e.g., conference poster, conference abstract, book chapter, comment, etc.); the paper was a review article; or the paper was not written in English.

### Data extraction and analysis

We extracted information regarding three main aspects: a) the healthcare context for AI fairness research, including healthcare datasets, data types, bias-relevant attributes, and involved clinical specialty; b) the techniques of AI fairness developed or applied in healthcare applications, including specific fairness definitions, bias identification, bias evaluation, and bias mitigation; and c) techniques that can enhance AI fairness, such as XAI, clinician-in-the-loop, and FL.

To clarify the topic of fairness, the grouping of fairness methods was organized into three categories, including pre-process, in-process, and post-process, according to Mehrabi et al^122^. and Xu et al.^27^, as shown in Box 1. Building on the general concepts of AI bias and fairness, we classified fairness into three categories to capture quantitative perspectives in AI fairness research^114^, drawing on the quantitative framework proposed by Balakrishnan et al^123^. These three categories are: group fairness, which dictates equal model results between subgroups separated by bias-relevant attributes; individual fairness, which encodes the notion that comparable individuals should be treated equally; and distribution fairness, which addresses fair distribution of limited resources (e.g., vaccination) across multiple stakeholders that can be both individuals and groups. We categorized existing fairness metrics, which address equality concerning various aspects of model decision-making, into these three definitions, with descriptions and examples in Box 1. For example, equalized odds^48,52^, which emphasizes the equality of model performance (sensitivity and specificity) across subgroups stratified by the bias-relevant attributes, is classified as a performance-based fairness metric that addresses group fairness.

We also extracted the algorithms used in each paper, referring to the AI model for bias identification, evaluation, and mitigation. Algorithms were classified into five categories, including traditional machine learning (e.g., linear models, SVM, tree-based models), discriminative DL models, GenAI, LLM, and others. Although LLM can be a subset of GenAI, we treated them as a distinct category due to their unique characteristics. We then analyzed the relationship between the algorithm types with bias identification, evaluation, and mitigation methods.

To visually report the results, descriptive summaries utilizing counts and proportions and figures such as evidence maps with marginal bar plots were reported, along with narrative interpretation when appropriate. R version 4.0.2 (The R Foundation for Statistical Computing) was used for data analysis.

## Supplementary information

Supplementary Information

## Data Availability

All data generated and analyzed during this study are available on OSF and can be accessed via https://osf.io/q5k2b/.

## Code availability

The codes for data analysis are available at the GitHub repository and can be accessed via https://github.com/nliulab/seeEvidenceGap.
