# Supplementary material for: A scoping review and evidence gap analysis of clinical AI fairness
Source: NPJ Digit Med. 2025 Jun 14;8:360. doi: 10.1038/s41746-025-01667-2 (PMC12167363; doi:10.1038/s41746-025-01667-2)
Supplement: Supplementary file 1 — Supplementary Information [file 41746_2025_1667_MOESM1_ESM.pdf]

Supplementary Materials

Table of Contents

Supplementary Figure 1. The evidence gap analysis of fairness metrics, cross-tabulated by high-level categories based on the fairness notions (group fairness, individual fairness and distributive fairness) and algorithm types. 1

Supplementary Table 1. List of public datasets stratified by data type and ordered by frequency in this review 2

Supplementary Table 2. Distribution of AI research among medical fields as documented by published systematic reviews 14

Supplementary Table 3. PRISMA-ScR Checklist 16

Supplementary Table 4. Search strategies 18

Supplementary Reference 19

Supplementary Figure 1. The evidence gap analysis of fairness metrics, cross-tabulated by high-level categories based on the fairness notions (group fairness, individual fairness and distributive fairness) and algorithm types.

		DL	ML	GenAI	LLM	Others	
**Group Fairness** Expect equal results across subgroups	**Parity–Based** Equal predicted positive rates across subgroups	51	65	17	9	20	134
	**Performance–Based** Equal model performance across subgroups	208	147	20	18	15	346
	**Rank–Based** Consistent score–based rankings across subgroups	3	5	1	0	1	9
	**Remove–Based** Remove bias–related attributes or confounders	4	2	2	0	0	7
	**Others–Based (Group)** E.g., measuring differences in subgroup data profiles, etc.	1	2	0	1	2	5
**Individual Fairness** Expect equal results for comparable individuals	**Counterfactual–Based** Invariance of decision–making against changes in bias–related attributes	3	4	0	1	1	7
	**Similarity–Based** Similar results for similar individuals	3	4	0	0	1	8
	**Others–Based (Individual)** E.g., de–diversity in individual gains, etc.	3	3	0	0	0	5
**Distribution Fairness** Expect fair distribution of limited resources	**Reward–Based** Reward resources based on contribution	5	2	0	0	0	7
	**Variance–Based** Equal resources received by participants	10	1	3	0	1	13

Each unit ("1") represents a single paper. One paper may involve multiple fairness notions and metrics. We did not observe obvious differences of bias evaluation metrics across algorithm types.

Supplementary Table 1. List of public datasets stratified by data type and ordered by frequency in this review

Data type	Public dataset	Medical fields[1]	Bias-relevant attributes	Number of papers
Tabular Static	MIMIC-III, MIMIC-IV	Cardiology, CC, ED, Geriatrics, ID, Neurology, Orthopedics, Pharmacy, Pulmonary, Renal, Surgery	Age, Education, Ethnicity/Race, Gender/Sex, Language, Lifestyle, Marital status, Socioeconomics, Not Specified	24
Tabular Static	MEPS	Informatics & Policy, Oral Health	Age, Ethnicity/Race, Gender/Sex, Socioeconomics, Not Specified	13
Tabular Static	Heritage Health	Informatics & Policy	Age, Gender/Sex, Not Specified	11
Tabular Static	UCI Heart Disease	Cardiology	Age, Gender/Sex	8
Tabular Static	SEER	Cancer, Gastroenterology, Hepatology, Pathology, Pulmonary, Urology	Age, Ethnicity/Race, Gender/Sex, Socioeconomics	7
Tabular Static	SUPPORT	Cancer, Cardiology, CC, Gastroenterology, Hepatology, ID, Neurology, Pulmonary	Age, Ethnicity/Race, Gender/Sex, Not Specified	6
Tabular Static	UCI diabetes	Cardiology, Endocrinology	Age, Education, Ethnicity/Race, Gender/Sex, Not Specified	6
Tabular Static	eICU	CC, Geriatrics	Age, Ethnicity/Race, Gender/Sex, Institute, Language	5
Tabular Static	TCGA	Cancer, Pathology, Renal	Age, Ethnicity/Race, Gender/Sex	4
Tabular Static	UCI Drug Consumption	Mental	Ethnicity/Race, Gender/Sex	4
Tabular Static	BRFSS	Cardiology, Endocrinology, Informatics & Policy, Public	Ethnicity/Race	3
Tabular Static	FLChain	Public	Age, Gender/Sex	3
Tabular Static	Framingham	Cardiology, Neurology	Ethnicity/Race, Gender/Sex, Not Specified	3
Tabular Static	UK Biobank	Endocrinology, Genetics, Mental, Primary care	Ethnicity/Race, Gender/Sex, Health conditions, Lifestyle, Socioeconomics	3
Tabular Static	All of Us	Cardiology, CC, Neurology	Age, Education, Ethnicity/Race, Gender/Sex, Lifestyle, Socioeconomics	2
Tabular Static	Arrhythmia	Cardiology	Gender/Sex	2
Tabular Static	Breast Cancer Wisconsin	Cancer	Not Specified	2
Tabular Static	Diabetic Hospital Dataset	Endocrinology	Age, Ethnicity/Race, Gender/Sex, Not Specified	2

Tabular Static	Integrated Data Repository	ID, Renal	Age, Ethnicity/Race, Gender/Sex, Socioeconomics	2
Tabular Static	MESA	Neurology	Ethnicity/Race, Not Specified	2
Tabular Static	MICD	Informatics & Policy	Gender/Sex, Not Specified	2
Tabular Static	NIS	Cancer, Cardiology	Ethnicity/Race, Location, Socioeconomics	2
Tabular Static	OAI	Anesthesiology, Orthopedics, Radiology, Rheumatology	Age, Anthropometry, Education, Ethnicity/Race, Gender/Sex, Socioeconomics	2
Tabular Static	Twitter	O & G	Ethnicity/Race	2
Tabular Static	130-US Hospitals84	Cardiology	Ethnicity/Race	1
Tabular Static	2020 NSDUH survey data	Mental, Pediatrics, Public	Ethnicity/Race, Gender/Sex, Lifestyle, Marital status, Socioeconomics	1
Tabular Static	ABCD	Neurology, Pediatrics, Radiology	Ethnicity/Race	1
Tabular Static	ABIDE	Neurology, Radiology	Age, Ethnicity/Race, Gender/Sex	1
Tabular Static	ACSPublicCoverage	Informatics & Policy	Gender/Sex	1
Tabular Static	ADNI	Geriatrics, Neurology	Gender/Sex	1
Tabular Static	ARIC	Neurology	Ethnicity/Race	1
Tabular Static	AURIN-PHA	Mental	Location	1
Tabular Static	American College of Surgeons Trauma Quality Improvement Program	PM & R, Surgery	Ethnicity/Race	1
Tabular Static	American Family Cohort	Informatics & Policy	Ethnicity/Race	1
Tabular Static	AmsterdamUMCdb	CC, Geriatrics	Age, Ethnicity/Race, Gender/Sex	1
Tabular Static	Atherosclerosis Risk in Communities (ARIC)	Neurology	Not Specified	1
Tabular Static	BH	ID, Pulmonary	Ethnicity/Race, Institute	1
Tabular Static	CBSA	Mental	Ethnicity/Race	1
Tabular Static	CDC 500 Cities data	Endocrinology, Public	Age, Ethnicity/Race, Socioeconomics	1
Tabular Static	CHESS	CC, ID	Age, Ethnicity/Race, Gender/Sex	1
Tabular Static	CPRD Covid-19 Synthetic datasets	ID	Ethnicity/Race, Gender/Sex, Location	1

Tabular Static	CRDC survey	Mental	Ethnicity/Race	1
Tabular Static	Cardiovascular disease dataset	Cardiology	Gender/Sex	1
Tabular Static	Cervical Dataset	Cancer, Neurology	Not Specified	1
Tabular Static	China Migrants Dynamic Survey	Informatics & Policy	Socioeconomics	1
Tabular Static	Clinical Practice Research Datalink	Cardiology	Ethnicity/Race, Gender/Sex, Location	1
Tabular Static	CoRDaCo dataset	ID, Informatics & Policy	Age, Ethnicity/Race, Gender/Sex, Location	1
Tabular Static	Coswara	ID, Public	Age, Ethnicity/Race, Gender/Sex, Health conditions, Language, Location, Time	1
Tabular Static	FAERS	Pharmacy	Gender/Sex	1
Tabular Static	FDA medical devices database	Eye	Ethnicity/Race	1
Tabular Static	FOS	Neurology	Ethnicity/Race	1
Tabular Static	FUUS	Mental, Pediatrics, Primary care, Public	Gender/Sex, Location	1
Tabular Static	GapMap	Mental	Ethnicity/Race	1
Tabular Static	Geisinger	ID, Public	Age, Ethnicity/Race, Gender/Sex, Socioeconomics	1
Tabular Static	Glima grading clinical and mutation feature	Cancer, Neurology	Ethnicity/Race	1
Tabular Static	HECKTOR 2022 challenge dataset	Cancer, Otolaryngology, Radiology	Age, Gender/Sex, Health conditions	1
Tabular Static	Harvard-FairVLMed	Eye	Ethnicity/Race, Gender/Sex, Language	1
Tabular Static	Harvard-GF	Eye	Ethnicity/Race, Gender/Sex	1
Tabular Static	Health Facts database	Endocrinology	Gender/Sex	1
Tabular Static	Health and Retirement Study	Geriatrics	Age, Ethnicity/Race, Gender/Sex, Location	1
Tabular Static	IBM MarketScan Medicaid Database	Mental, O & G	Ethnicity/Race	1
Tabular Static	IHDP	Pediatrics	Ethnicity/Race	1
Tabular Static	IWPC dataset	Pharmacy	Ethnicity/Race	1
Tabular Static	Indian Liver Patient Dataset (ILPD)	Hepatology	Gender/Sex	1
Tabular Static	Infections in Oxfordshire Research	ED, ID	Ethnicity/Race, Institute	1

	Database (IORD)			
Tabular Static	KiTS19	Cancer, Renal, Surgery, Urology	Age, Ethnicity/Race, Gender/Sex	1
Tabular Static	LONGSCAN	Mental, Pediatrics, Primary care, Public	Ethnicity/Race, Gender/Sex	1
Tabular Static	MAIKI	Mental	Gender/Sex	1
Tabular Static	MIDRC	ID, Pulmonary, Radiology	Age, Ethnicity/Race, Gender/Sex	1
Tabular Static	MMRF CoMMpass	Cancer, Pathology	Ethnicity/Race	1
Tabular Static	NHANES	Mental, Primary care, Public	Ethnicity/Race, Gender/Sex, Health conditions, Socioeconomics	1
Tabular Static	NIA Alzheimer's Disease Center's Cohort	Cancer, Genetics, Geriatrics, Pulmonary, Urology	Ethnicity/Race	1
Tabular Static	NPD-CRIS linkage data	Mental, Pediatrics	Ethnicity/Race, Language	1
Tabular Static	Nafld1	Hepatology, Public	Gender/Sex	1
Tabular Static	National Central Cancer Registry of China	Cancer, Gastroenterology	Gender/Sex, Location	1
Tabular Static	National Comorbidity Survey Replication Adolescent Supplement (NCS-A)	Mental	Ethnicity/Race, Gender/Sex	1
Tabular Static	National Health Interview Survey (NHIS) data (2020-2022) from the IPUMS12 and identified a cohort of children (ages 0-17).	ID, Public	Ethnicity/Race	1
Tabular Static	National Health and Aging Trends Study (NHATS)	Geriatrics, Neurology	Education, Ethnicity/Race	1
Tabular Static	National Lung Screening Trail (NLST)	Cardiology, Pulmonary, Radiology	Ethnicity/Race	1
Tabular Static	Non-Small Cell Lung Cancer (NSCLC) clinical trials	Cancer, Pulmonary	Age, Gender/Sex	1
Tabular Static	Northern Alberta Cancer Dataset (NACD)	Cancer	Not Specified	1

Tabular Static	Nursing Home Compare Star Ratings	Geriatrics, Public	Age, Ethnicity/Race, Gender/Sex, Socioeconomics	1
Tabular Static	OASIS	Neurology	Gender/Sex	1
Tabular Static	OPTN	Hepatology	Age, Ethnicity/Race, Gender/Sex	1
Tabular Static	OUH	ID, Pulmonary	Ethnicity/Race, Institute	1
Tabular Static	Older Adults	Geriatrics	Gender/Sex	1
Tabular Static	OncoArray	Cancer, Genetics, Geriatrics, Pulmonary, Urology	Ethnicity/Race	1
Tabular Static	Optum Labs Data Warehouse	Informatics & Policy	Ethnicity/Race	1
Tabular Static	Organ Procurement and Transplantation Network (OPTN)	Hepatology	Age, Ethnicity/Race, Gender/Sex	1
Tabular Static	Our world in Data	ID	Gender/Sex	1
Tabular Static	PHENOM	Neurology, Radiology	Age, Ethnicity/Race, Gender/Sex	1
Tabular Static	PIC43	Pulmonary	Ethnicity/Race	1
Tabular Static	PRAMS	O & G	Ethnicity/Race	1
Tabular Static	PRAp	Cancer, Urology	Ethnicity/Race, Socioeconomics	1
Tabular Static	PUH	ID, Pulmonary	Ethnicity/Race, Institute	1
Tabular Static	REGARDS	Neurology	Ethnicity/Race	1
Tabular Static	SIVEP-Gripe data	ID	Gender/Sex	1
Tabular Static	SPRINT	Cardiology	Ethnicity/Race, Gender/Sex	1
Tabular Static	SisPorto 2.0	Cardiology, O & G	Gender/Sex	1
Tabular Static	Stanford Medicine Research Data Repository	Cardiology	Age, Ethnicity/Race, Gender/Sex	1
Tabular Static	Starter	Endocrinology	Ethnicity/Race	1
Tabular Static	Statistics NZ Integrated Data Infrastructure (IDI)	O & G	Age, Education, Ethnicity/Race, Location, Socioeconomics	1
Tabular Static	Stroke Dataset	Cancer, Neurology	Not Specified	1
Tabular Static	Systolic Blood Pressure Intervention Trial (SPRINT) with NHANES	Cardiology	Age, Education, Ethnicity/Race, Gender/Sex	1

Tabular Static	TARGET	Cancer, Pathology	Ethnicity/Race	1
Tabular Static	The Global health 50/50	ID	Gender/Sex	1
Tabular Static	The Standard Transparent Analysis and Research (STAR) organ transplant dataset	CC, Hepatology	Ethnicity/Race, Gender/Sex	1
Tabular Static	Tumor	Cancer, Gastroenterology	Gender/Sex	1
Tabular Static	UCI ASD	Mental	Gender/Sex	1
Tabular Static	UCI Cardiovascular Disease	Cardiology	Age, Gender/Sex	1
Tabular Static	UCI Obesity	Public	Gender/Sex	1
Tabular Static	UCI Parkinsons Telemonitoring	Geriatric, Neurology	Gender/Sex	1
Tabular Static	UHB	ID, Pulmonary	Ethnicity/Race, Institute	1
Tabular Static	UK Adult ITP Registry (UKITPR)	Hematology, Immunology	Age, Ethnicity/Race, Gender/Sex	1
Tabular Static	UK ME/CFS Biobank (UKMEB)	Endocrinology, Mental	Gender/Sex	1
Tabular Static	United Nation database	O & G, Pediatrics	Clinician expertise, Gender/Sex, Location	1
Tabular Static	VHA Corporate Data Warehouse	Mental	Ethnicity/Race, Gender/Sex	1
Tabular Static	Victorian minimum emergency dataset (VEMD)	ED	Gender/Sex, Location	1
Tabular Static	WHAS	Cardiology	Gender/Sex	1
Tabular Static	dbGaP datasets	Cancer, Genetics, Geriatrics, Pulmonary, Urology	Ethnicity/Race	1
Tabular Static	iSTAGING	Neurology, Radiology	Age, Ethnicity/Race, Gender/Sex	1
Tabular Static	rRNA gene sequences datasets	ID, O & G, Pathology	Ethnicity/Race	1
Tabular Static	the OCT equipment user manual of the following contemporary OCT equipment	Eye	Ethnicity/Race	1
Tabular Temporal	MIMIC-III, MIMIC-IV	CC, ID	Age, Ethnicity/Race, Gender/Sex, Language, Marital status, Socioeconomics	9

Tabular Temporal	MEPS	Informatics & Policy	Ethnicity/Race, Gender/Sex	2
Tabular Temporal	Optum CDM	Cancer, Informatics & Policy, Pulmonary	Age, Ethnicity/Race, Gender/Sex	2
Tabular Temporal	SEER	Cancer, Pulmonary	Age, Ethnicity/Race	2
Tabular Temporal	STARR	CC, Informatics & Policy	Age, Ethnicity/Race, Gender/Sex, Socioeconomics	2
Tabular Temporal	2019-nCoV	ED, ID	Location	1
Tabular Temporal	ACS 5-year	Public	Ethnicity/Race, Socioeconomics	1
Tabular Temporal	ARIC (Atherosclerosis Risk in Communities Study)	Cardiology	Ethnicity/Race, Gender/Sex	1
Tabular Temporal	ATUS	Public	Age, Gender/Sex	1
Tabular Temporal	CARDIA (Coronary Artery Risk Development in Young Adults Study)	Cardiology	Ethnicity/Race, Gender/Sex	1
Tabular Temporal	CHS (Cardiovascular Health Study 1989)	Cardiology	Ethnicity/Race, Gender/Sex	1
Tabular Temporal	CirCor DigiScope Phonocardiogra m Dataset	Cardiology, Pediatrics	Gender/Sex	1
Tabular Temporal	FHS OS (Framingham Heart Study Offspring Cohort)	Cardiology	Ethnicity/Race, Gender/Sex	1
Tabular Temporal	JHS (Jackson Heart Study)	Cardiology	Ethnicity/Race, Gender/Sex	1
Tabular Temporal	MESA	Cardiology	Ethnicity/Race, Gender/Sex	1
Tabular Temporal	MIDRC	ID	Age, Ethnicity/Race, Gender/Sex	1
Tabular Temporal	NCDB	Urology	Ethnicity/Race	1
Tabular Temporal	NSQIP	Orthopedics, Surgery	Ethnicity/Race, Gender/Sex	1
Tabular Temporal	National Health and Nutrition Examination Survey	Public	Age	1
Tabular Temporal	Scientific Registry of Transplant Recipients (SRTR)	Immunology, Renal	Ethnicity/Race	1

Tabular Temporal	UK Biobank	Cardiology, Endocrinology, Pathology	Age, Ethnicity/Race, Gender/Sex	1
Tabular Temporal	World bank data	O & G, Pediatrics	Gender/Sex	1
Tabular Temporal	eICU	CC	Ethnicity/Race	1
Image	ISIC (HAM10000, BCM20000, etc.)	Cancer, Dermatology, Radiology	Age, Ethnicity/Race, Gender/Sex, Health conditions, Skin tone, Not Specified	20
Image	CheXpert	Cardiology, Dermatology, Pulmonary, Radiology	Age, Ethnicity/Race, Gender/Sex, Skin tone	18
Image	Fitzpatrick17k	Cancer, Dermatology	Age, Gender/Sex, Health conditions, Skin tone	13
Image	MIMIC-CXR	Cardiology, Dermatology, Pulmonary, Radiology	Age, Ethnicity/Race, Gender/Sex, Skin tone, Socioeconomics	13
Image	Chest-Xray8, Chest-Xray14	Cardiology, ID, Pulmonary, Radiology	Age, Ethnicity/Race, Gender/Sex	12
Image	ADNI	Geriatrics, Neurology, Radiology	Age, Gender/Sex	5
Image	EyePACS	Endocrinology, Eye	Age, Ethnicity/Race, Gender/Sex, Health conditions, Skin tone, Not Specified	5
Image	TCGA	Cancer, Pathology, Pulmonary, Renal	Age, Ethnicity/Race, Institute, Socioeconomics	4
Image	ABIDE	Mental, Neurology, Pediatrics, Radiology	Age, Ethnicity/Race, Gender/Sex, Health conditions, Lifestyle, Others	3
Image	DDI	Cancer, Dermatology	Age, Ethnicity/Race, Skin tone	3
Image	MIDRC	ID, Pulmonary, Radiology	Age, Ethnicity/Race, Gender/Sex	3
Image	OAI	Anesthesiology, Orthopedics, Radiology, Rheumatology	Education, Ethnicity/Race, Gender/Sex, Socioeconomics	3
Image	UK Biobank CMR images	Cardiology, Radiology	Ethnicity/Race, Gender/Sex	3
Image	ABCD	Neurology, Pediatrics, Radiology	Ethnicity/Race	2
Image	CelebA	Dermatology, Informatics & Policy	Health conditions, Skin tone	2
Image	Covid Chestxay	ID, Pathology, Pulmonary, Radiology	Age, Gender/Sex	2
Image	Dermnet	Cancer, Dermatology	Skin tone	2
Image	HCP	Neurology, Radiology	Ethnicity/Race, Gender/Sex	2
Image	IQ-OTH/NCCD	Cancer, Pulmonary, Radiology	Not Specified	2
Image	JSRT	Cardiology, ID, Pulmonary, Radiology	Age, Ethnicity/Race, Gender/Sex	2
Image	PAD-UFES-20	Cancer, Dermatology	Skin tone	2

Image	PathMNIST	Cancer, Gastroenterology, Pathology	Not Specified	2
Image	AIM-Ahead	Cancer	Ethnicity/Race	1
Image	APTOS	Endocrinology, Eye	Not Specified	1
Image	AREDS	Eye, Geriatrics	Age, Ethnicity/Race, Gender/Sex	1
Image	Brain Tumour Segmentation (BraTS) 2019 challenge dataset	Cancer, Neurology, Pathology	Health conditions	1
Image	BreastScreen Reader Assessment Strategy Australia	Cancer, Public, Radiology	Not Specified	1
Image	Brixia COVID-19 dataset	ID, Pulmonary, Radiology	Age, Gender/Sex	1
Image	CAMELYON17	Pathology	Institute	1
Image	COVID-19 Chest X-rays (NY-SBU)	ID, Pulmonary, Radiology	Age, Gender/Sex	1
Image	Calgary-Campinas-359 (CC359)	Neurology, Radiology	Gender/Sex	1
Image	Chest CT-Scan	Cancer, Pulmonary, Radiology	Not Specified	1
Image	Cohort of Screen-age Women - Case-control	Cancer, Public, Radiology	Not Specified	1
Image	Covid-19 Chest X-rays for Mortality Prediction	ID, Pulmonary, Radiology	Age, Gender/Sex	1
Image	Covid19	ID	Institute, Not Specified	1
Image	Dermofit dataset	Cancer, Dermatology	Skin tone	1
Image	EBRAINS brain tumor atlas	Cancer, Neurology, Pathology	Age, Ethnicity/Race	1
Image	ESFair contest	Dermatology	Not Specified	1
Image	Emory-CXR	Pulmonary, Radiology	Age, Ethnicity/Race, Gender/Sex	1
Image	FRGCv2	Informatics & Policy	Health conditions	1
Image	GestaltMatcher Database	Informatics & Policy	Age, Ethnicity/Race, Gender/Sex	1
Image	Guangzhou pediatric dataset	Pediatrics, Pulmonary, Radiology	Age	1
Image	HECKTOR 2022 challenge dataset	Cancer, Otolaryngology, Radiology	Age, Gender/Sex, Health conditions	1
Image	Harvard-FairVLMed	Eye	Ethnicity/Race, Gender/Sex, Language	1
Image	Harvard-GF	Eye	Ethnicity/Race, Gender/Sex	1

Image	Human Connectome Project	Neurology	Gender/Sex	1
Image	Kaggle 2020 Melanoma Competition dataset	Cancer, Dermatology	Skin tone	1
Image	KiTS 2019 dataset	Cancer, Radiology, Renal	Age, Gender/Sex	1
Image	London Medical Database	Informatics & Policy	Age, Ethnicity/Race, Gender/Sex	1
Image	MINIST	Informatics & Policy	Institute	1
Image	MixNAF	Eye	Age, Gender/Sex	1
Image	MoDL-Brain	Neurology, Radiology	Not Specified	1
Image	Montgomery	Cardiology, Pulmonary, Radiology	Gender/Sex	1
Image	Mpox Skin Lesion Dataset Version 2.0 (MSLD v2.0)	Dermatology, ID	Ethnicity/Race, Skin tone	1
Image	National Genome Research Network Plus	Mental	Age, Gender/Sex	1
Image	National Health and Aging Trends Study (NHATS)	Geriatrics, Neurology	Education, Ethnicity/Race	1
Image	OASIS	Neurology	Gender/Sex	1
Image	ODIR	Eye	Age, Gender/Sex	1
Image	OHTS	Eye	Age, Ethnicity/Race, Gender/Sex	1
Image	OPTIMAM Mammography Imaging Database	Cancer, Public, Radiology	Age, Ethnicity/Race, Health conditions	1
Image	OculoScope	Eye	Age, Gender/Sex	1
Image	Open Access Series of Imaging Studies	Neurology, Radiology	Age, Gender/Sex	1
Image	PAPILA	Eye	Age, Gender/Sex	1
Image	PH2	Cancer, Dermatology	Skin tone	1
Image	PHENOM	Neurology, Radiology	Age, Ethnicity/Race, Gender/Sex	1
Image	PadChest	Cardiology, Pulmonary, Radiology	Age, Ethnicity/Race, Gender/Sex	1
Image	SIIM	Cardiology, Pulmonary, Radiology	Age, Ethnicity/Race, Gender/Sex	1
Image	Shenzhen	Cardiology, Pulmonary, Radiology	Gender/Sex	1
Image	Shenzhen chest X-ray set	ID, Pulmonary, Radiology	Age, Ethnicity/Race	1
Image	The Chinese Mammography Dataset	Cancer, Public, Radiology	Not Specified	1
Image	The Mobile Ocular	Eye	Ethnicity/Race, Others	1

	Biometrics In Unconstrained Settings			
Image	The Mpox Close Skin Images (MCSI) dataset	Dermatology, ID	Skin tone	1
Image	The Multi-Angle Sclera Dataset	Eye	Ethnicity/Race, Others	1
Image	The Sclera Blood Vessels Periocular and Iris	Eye	Ethnicity/Race, Others	1
Image	The Sclera Liveness Dataset	Eye	Ethnicity/Race, Others	1
Image	The Sclera Mobile Dataset	Eye	Ethnicity/Race, Others	1
Image	UCLA Consortium dataset	Mental, Radiology	Gender/Sex, Health conditions	1
Image	UCSF-PDGM	Cancer, Neurology, Radiology	Age, Gender/Sex	1
Image	UK biobank	Cardiology, Radiology	Ethnicity/Race, Gender/Sex	1
Image	UPenn-GBM	Cancer, Neurology, Radiology	Age, Gender/Sex	1
Image	VinDr	Cardiology, Pulmonary, Radiology	Age, Ethnicity/Race, Gender/Sex	1
Image	Waterloo dataset	Cancer, Dermatology	Skin tone	1
Image	a minor subset of the Padchest dataset	Cardiology, Pulmonary, Radiology	Gender/Sex	1
Image	cc359	Neurology, Radiology	Not Specified	1
Image	chest-xray-pneumonia	Pulmonary, Radiology	Not Specified	1
Image	fastMRI	Neurology, Radiology	Not Specified	1
Image	iSTAGING	Neurology, Radiology	Age, Ethnicity/Race, Gender/Sex	1
Image	the Cambridge Centre for Ageing and Neuroscience (CamCAN)	Neurology, Radiology	Gender/Sex	1
Image	the Radiological Hand Pose Estimation (RHPE) dataset	Pediatrics, Radiology	Age, Ethnicity/Race, Gender/Sex	1
Image	the Radiology Society of North America (RSNA) dataset	Pediatrics, Radiology	Age, Ethnicity/Race, Gender/Sex	1
Video	UBFC-RPPG	Informatics & Policy	Skin tone	1
Video	UCLA-rPPG	Hematology	Skin tone	1
Video	VITAL	Informatics & Policy	Skin tone	1
Signal	1000 fragments	Cardiology	Age, Gender/Sex	1
Signal	2021 PhysioNet/Computing in	Cardiology	Age, Ethnicity/Race, Gender/Sex	1

	Cardiology Challenge			
Signal	Arrhythmia	Cardiology	Age, Gender/Sex	1
Signal	Clinical TUH Abnormal EEG Corpus	Neurology	Gender/Sex	1
Signal	D-Vlog	Mental	Gender/Sex	1
Signal	DAIC-WoZ	Mental	Ethnicity/Race, Gender/Sex	1
Signal	Depresjon	Mental	Age, Gender/Sex	1
Signal	IntelliRehab (IRDS)	Neurology, PM & R	Ethnicity/Race, Gender/Sex, Health conditions	1
Signal	Psykose	Mental	Age, Gender/Sex	1
Signal	Tiles-2018	Mental	Age, Ethnicity/Race, Gender/Sex, Institute, Language, Lifestyle, Location, Socioeconomics	1
Signal	UBFC-RPPG	Informatics & Policy	Skin tone	1
Signal	VITAL	Informatics & Policy	Skin tone	1
Signal	VerBIo	Mental	Ethnicity/Race, Gender/Sex	1
Audio	ADReSS challenge dataset	Neurology	Not Specified	1
Audio	DAIC-WOZ	Mental	Gender/Sex	1
Text	MIMIC-III, MIMIC-IV	CC, Mental	Ethnicity/Race, Gender/Sex, Not Specified	6
Text	Twitter	Mental, O & G	Ethnicity/Race, Location	3
Text	AskAPatient	Mental	Age, Gender/Sex	1
Text	FluTrack	ID, Public	Ethnicity/Race	1
Text	FluVacc	ID, Public	Ethnicity/Race	1
Text	Harvard-FairVLMed	Eye	Ethnicity/Race, Gender/Sex, Language	1
Text	HealthQA	Informatics & Policy	Language	1
Text	LiveQA	Informatics & Policy	Language	1
Text	Medical transcription dataset(mtsampl es.com)	Informatics & Policy	Gender/Sex	1
Text	MedicationQA	Informatics & Policy	Language	1
Text	Psychometric	Mental	Age, Education, Ethnicity/Race, Gender/Sex, Socioeconomics	1
Text	U.S. Census and U.S. Social Security	Informatics & Policy	Ethnicity/Race, Gender/Sex, Others, Time	1

[1]refers to those medical fields where the databases were applied as observed in this review, rather than how these databases are defined.

Supplementary Table 2. Distribution of AI research among medical fields as documented by published systematic reviews

**Medical fields**	**Number of AI research included**	**Databases**	**Review**	**Search up to year**	**Note[1]**
Informatics & Policy	/	/	/	/	
Cancer	3391	Medline	Suero-Abreu et al., 2022[1]	2019	
Radiology	11209	Web of Science	Kocak et al., 2023[2]	(2000-) 2021	Bibliometric analysis
Cardiology	1172	Medline	Suero-Abreu et al., 2022[1]	2019	
ID (Infectious Disease)	624	Medline	Rabaan et al., 2023[3]	2022	
Mental Health	300	PsycInfo, Cochrane, Medline, IEEE, ACM	Shatte et al., 2019[4]	2018	
CC (Critical Care)	494	Embase, Medline, Web of science, Cochrane, Google scholar	Van de Sande et al., 2021[5]	2020	
Neurology	155	Medline, Scopus, Web of Science	Segato et al., 2020[6]	2020	
Pulmonology	156	Medline, DBLP	Exarchos et al., 2021[7]	2020	Chronic Obstructive Lung Disease
Public Health	/	/	/	/	
Pediatric	363	Medline, Cochrane, the Cumulative Index to Nursing and Allied Health Literature Plus, Web of Science, EBSCO Dentistry & Oral Science Source	Hoodbhoy et al., 2021[8]	2020	
Dermatology	143	Medline, Web of Science, Google scholar, CQVIP, Wanfang Data, CNKI	Li et al., 2022[9]	2022	Dermatology image
ED (Emergency Department)	395	Medline-OVID, Embase, CINAHL, IEEE	Kirubarajan et al., 2020[10]	2020	
Endocrinology	17	Medline	Giorgini et al., 2023[11]	2023	
O & G (Obstetrics and Gynecology)	66	Medline	Dhombres et al., 2022[12]	2020	
Eye	69	Medline, Scopus	Nuzzi et al., 2021[13]	2021	
Gastroenterology	73	Medline, CINAHL, Cochrane, Web of Science	Parkash et al., 2022[14]	2021	Gastrointestinal luminal pathologies
Geriatrics	105	Medline, EBASE, Cochrane, Web of Science, PyscInfo, CNKI, SinoMed, WANFANG, VIP	Ma et al., 2023[15]	2023	

Hepatology	150	Medline	Balsano et al., 2022[16]	2022	
Pharmacy	/	Medline, Google Scholar, Scopus	Chalasani et al., 2023[17]	2023	Numbers not reported
Surgery	/	/	Varghese et al., 2024[18]	2024	number and databases was not reported
Orthopedics	223	Embase, Medline, Scopus	Federer et al., 2021[19]	2019	
Pathology	100	Medline, Embase, CENTRAL	McGenity et al., 2024[20]	2024	
Urology	112	/	Chen et al., 2022[21]	2020	database was not reported
Otolaryngology	54	Medline	Bur et al., 2019[22]	2018	
Family Medicine	405	Medline-OVID, Embase, CINAHL, Cochrane, Web of Science, Scopus, IEEE, ACM Digital Library, MathSciNet, AAAI	Kueper et al., 2020[23]	2020	
Renal (Nephrology)	218	the Institute for Scientific Information Web of Knowledge database	Park et al., 2021[24]	2020	
Rheumatology	91	Medline, Scopus, Web of Science, Rheumatology journals	Madrid-García et al., 2023[25]	2021	
Anesthesiology	173	Medline, Embase, Web of Science, IEEE	Hashimoto et al., 2020[26]	2018	
Hematology	53	Medline	Kotsyfakis et al., 2022[27]	2022	Hematology oncology
Oral Health	178	Cochrane, Medline, Scopus, IEEE, Arxiv	Morch et al. 2021[28]	2020	
Immunology	169	Medline, Embase	Stafford et al., 2020[29]	2018	Autoimmune disease
Occupational Medicine	27	Medline, IEEE, Web of Science	Chaudhry et al, 2024[30]	2014-2024	
PM & R (Physical Medicine and Rehabilitation)	28	Medline, Embase, CINAHL, NARIC, Web of Science, OpenGrey	Sumner et al, 2023[31]	2021	

[1]When no systematic review exists for a specific medical field due to its broad nature, an alternative smaller field was used.

Supplementary Table 3. PRISMA-ScR Checklist

Section	Item	PRISMA-ScR checklist item	Reported on page #
**TITLE**			
Title	1	Identify the report as a scoping review.	1
**ABSTRACT**			
Structured summary	2	Provide a structured summary that includes (as applicable): background, objectives, eligibility criteria, sources of evidence, charting methods, results, and conclusions that relate to the review questions and objectives.	2
**INTRODUCTION**			
Rationale	3	Describe the rationale for the review in the context of what is already known. Explain why the review questions/objectives lend themselves to a scoping review approach.	3
Objectives	4	Provide an explicit statement of the questions and objectives being addressed with reference to their key elements (e.g., population or participants, concepts, and context) or other relevant key elements used to conceptualize the review questions and/or objectives.	4
**METHODS**			
Protocol and registration	5	Indicate whether a review protocol exists; state if and where it can be accessed (e.g., a Web address); and if available, provide registration information, including the registration number.	13
Eligibility criteria	6	Specify characteristics of the sources of evidence used as eligibility criteria (e.g., years considered, language, and publication status), and provide a rationale.	14
Information sources*	7	Describe all information sources in the search (e.g., databases with dates of coverage and contact with authors to identify additional sources), as well as the date the most recent search was executed.	13
Search	8	Present the full electronic search strategy for at least 1 database, including any limits used, such that it could be repeated.	13
Selection of sources of evidence†	9	State the process for selecting sources of evidence (i.e., screening and eligibility) included in the scoping review.	13-14
Data charting process‡	10	Describe the methods of charting data from the included sources of evidence (e.g., calibrated forms or forms that have been tested by the team before their use, and whether data charting was done independently or in duplicate) and any processes for obtaining and confirming data from investigators.	14-15
Data items	11	List and define all variables for which data were sought and any assumptions and simplifications made.	13-15, 29-30
Critical appraisal of individual sources of evidence§	12	If done, provide a rationale for conducting a critical appraisal of included sources of evidence; describe the methods used and how this information was used in any data synthesis (if appropriate).	NA

Section	Item	PRISMA-ScR checklist item	Reported on page #
Synthesis of results	13	Describe the methods of handling and summarizing the data that were charted.	14-15
**RESULTS**			
Selection of sources of evidence	14	Give numbers of sources of evidence screened, assessed for eligibility, and included in the review, with reasons for exclusions at each stage, ideally using a flow diagram.	4,25
Characteristics of sources of evidence	15	For each source of evidence, present characteristics for which data were charted and provide the citations.	4-9
Critical appraisal within sources of evidence	16	If done, present data on critical appraisal of included sources of evidence (see item 12).	NA
Results of individual sources of evidence	17	For each included source of evidence, present the relevant data that were charted that relate to the review questions and objectives.	4-9
Synthesis of results	18	Summarize and/or present the charting results as they relate to the review questions and objectives.	4-9
**DISCUSSION**			
Summary of evidence	19	Summarize the main results (including an overview of concepts, themes, and types of evidence available), link to the review questions and objectives, and consider the relevance to key groups.	9-13
Limitations	20	Discuss the limitations of the scoping review process.	13
Conclusions	21	Provide a general interpretation of the results with respect to the review questions and objectives, as well as potential implications and/or next steps.	13
**FUNDING**			
Funding	22	Describe sources of funding for the included sources of evidence, as well as sources of funding for the scoping review. Describe the role of the funders of the scoping review.	15

Supplementary Table 4. Search strategies

Database	Search strategy
MEDLINE	(("machine learning") OR ("deep learning") OR ("artificial intelligence")) AND ("fair*"[tiab] OR "equality"[tiab] OR "equity"[tiab] OR ((“alleviat*”[tiab] OR"mitigat*"[tiab] OR "reduc*"[tiab] OR "eliminat*"[tiab] OR "evaluat*"[tiab] OR "assess*"[tiab] OR "measure*"[tiab]OR "detect*"[tiab]) NEAR ("bias*"[tiab] OR "disparit*"[tiab] OR "inequality"[tiab] OR “inequity”[tiab]))) AND (("health*") OR ("medicine") OR ("clinical") OR ("medical"))
Web of Science	ALL=(("machine learning") OR ("deep learning") OR ("artificial intelligence")) AND AB=("fair*” OR “equality” OR “equity” OR ((“alleviat*” OR “mitigat*” OR "reduc*" OR “eliminat*” OR “evaluat*” OR “assess*” OR “measure*” OR “detect*”) NEAR (“bias*” OR “disparit*” OR “inequality” OR “inequity”))) AND ALL=(("health*") OR ("medicine") OR ("clinical") OR ("medical"))
Embase	('machine learning' OR 'deep learning' OR 'artificial intelligence') AND (‘fair*’:ab,ti OR ‘equality’:ab,ti OR ‘equity’:ab,ti OR ((‘alleviat*’ OR ‘mitigat*’ OR ‘reduc*’ OR ‘eliminat*’ OR ‘evaluat*’ OR ‘assess*’ OR ‘measure*’ OR ‘detect*’) NEAR/15 (‘bias*’ OR ‘disparit*’ OR ‘inequality’ OR ‘inequity’)):ab,ti) AND ('health*' OR 'medicine' OR 'clinical' OR 'medical')
Scopus	(("machine learning") OR ("deep learning") OR ("artificial intelligence")) AND TITLE-ABS-KEY (("fair*" OR "equality" OR "equity" OR (("alleviat*" OR "mitigat*" OR "reduc*" OR "eliminat*" OR "evaluat*" OR "assess*" OR "measure*" OR "detect*") near/15 ("bias*" OR "disparit*" OR "inequality" OR "inequity")))) AND (("health*") OR ("medicine") OR ("clinical") OR ("medical"))
IEEE Xplore	((("Full Text & Metadata":"machine learning" OR "Full Text & Metadata":"deep learning" OR "Full Text & Metadata":"artificial intelligence") AND ("Abstract":"fair" OR "Abstract":"fairness" OR "Abstract":"fairly" OR "Abstract":"equality" OR "Abstract":"equity" OR (("Abstract":“alleviat*” OR "Abstract":"mitigat*" OR "Abstract":"reduc*" OR "Abstract":"eliminat*" OR "Abstract":"evaluat*" OR "Abstract":"assess*" OR "Abstract":"measure*"OR "Abstract":"detect*") NEAR ("Abstract":"bias" OR “Abstract”:”biases” OR "Abstract":"disparity" OR “Abstract”:”disparities” OR "Abstract":"inequality" OR

	“Abstract”:”inequity”))) AND ("Full Text & Metadata":"health*" OR "Full Text & Metadata":"medicine" OR "Full Text & Metadata":"clinical" OR "Full Text & Metadata":"medical")))
ACM Library	[[All: "machine learning"] OR [All: "deep learning"] OR [All: "artificial intelligence"]] AND [[Abstract: "fair*"] OR [Abstract: "equality"] OR [Abstract: "equity"] OR [[[Abstract: "alleviat*"] OR [Abstract: "mitigat*"] OR [Abstract: "reduc*"] OR [Abstract: "eliminat*"] OR [Abstract: "evaluat*"] OR [Abstract: "assess*"] OR [Abstract: "measure*"] OR [Abstract: "detect*"]] AND [[Abstract: "bias*"] OR [Abstract: "disparit*"] OR [Abstract: "inequality"]]]] AND [[All: "health*"] OR [All: "medicine"] OR [All: "clinical"] OR [All: "medical"]]

Supplementary Reference

1 Suero-Abreu, G. A., Hamid, A., Akbilgic, O. & Brown, S.-A. Trends in cardiology and oncology artificial intelligence publications. *American Heart Journal Plus: Cardiology Research and Practice* **17**, 100162 (2022). https://doi.org:https://doi.org/10.1016/j.ahjo.2022.100162

2 Kocak, B., Baessler, B., Cuocolo, R., Mercaldo, N. & Pinto dos Santos, D. Trends and statistics of artificial intelligence and radiomics research in Radiology, Nuclear Medicine, and Medical Imaging: bibliometric analysis. *European Radiology* **33**, 7542-7555 (2023). https://doi.org:10.1007/s00330-023-09772-0

3 Rabaan, A. A. *et al.* Unleashing the power of artificial intelligence for diagnosing and treating infectious diseases: A comprehensive review. *Journal of Infection and Public Health* **16**, 1837-1847 (2023). https://doi.org:https://doi.org/10.1016/j.jiph.2023.08.021

4 Shatte, A. B. R., Hutchinson, D. M. & Teague, S. J. Machine learning in mental health: a scoping review of methods and applications. *Psychol Med* **49**, 1426-1448 (2019). https://doi.org:10.1017/s0033291719000151

5 van de Sande, D., van Genderen, M. E., Huiskens, J., Gommers, D. & van Bommel, J. Moving from bytes to bedside: a systematic review on the use of artificial intelligence in the intensive care unit. *Intensive Care Medicine* **47**, 750-760 (2021). https://doi.org:10.1007/s00134-021-06446-7

6 Segato, A., Marzullo, A., Calimeri, F. & De Momi, E. Artificial intelligence for brain diseases: A systematic review. *APL Bioengineering* **4**, 041503 (2020). https://doi.org:10.1063/5.0011697

7 Exarchos, K. P. *et al.* Review of Artificial Intelligence Techniques in Chronic Obstructive Lung Disease. *IEEE Journal of Biomedical and Health Informatics* **26**, 2331-2338 (2022). https://doi.org:10.1109/JBHI.2021.3135838

8 Hoodbhoy, Z. *et al.* Machine Learning for Child and Adolescent Health: A Systematic Review. *Pediatrics* **147**, e2020011833 (2021). https://doi.org:10.1542/peds.2020-011833

9 Li, Z., Koban, K. C., Schenck, T. L., Giunta, R. E., Li, Q. & Sun, Y. Artificial Intelligence in Dermatology Image Analysis: Current Developments and Future Trends. *Journal of Clinical Medicine* **11** (2022).

10 Kirubarajan, A., Taher, A., Khan, S. & Masood, S. Artificial intelligence in emergency medicine: A scoping review. *Journal of the American College of*

Emergency Physicians Open **1**, 1691-1702 (2020). https://doi.org:https://doi.org/10.1002/emp2.12277
11 Giorgini, F., Di Dalmazi, G. & Diciotti, S. Artificial intelligence in endocrinology: a comprehensive review. *J Endocrinol Invest* **47**, 1067-1082 (2024). https://doi.org:10.1007/s40618-023-02235-9
12 Dhombres, F., Bonnard, J., Bailly, K., Maurice, P., Papageorghiou, A. T. & Jouannic, J. M. Contributions of Artificial Intelligence Reported in Obstetrics and Gynecology Journals: Systematic Review. *J Med Internet Res* **24**, e35465 (2022). https://doi.org:10.2196/35465
13 Nuzzi, R., Boscia, G., Marolo, P. & Ricardi, F. The Impact of Artificial Intelligence and Deep Learning in Eye Diseases: A Review. *Front Med (Lausanne)* **8**, 710329 (2021). https://doi.org:10.3389/fmed.2021.710329
14 Parkash, O. *et al.* Diagnostic accuracy of artificial intelligence for detecting gastrointestinal luminal pathologies: A systematic review and meta-analysis. *Front Med (Lausanne)* **9**, 1018937 (2022). https://doi.org:10.3389/fmed.2022.1018937
15 Ma, B. *et al.* Artificial intelligence in elderly healthcare: A scoping review. *Ageing Res Rev* **83**, 101808 (2023). https://doi.org:10.1016/j.arr.2022.101808
16 Balsano, C. *et al.* The application of artificial intelligence in hepatology: A systematic review. *Digestive and Liver Disease* **54**, 299-308 (2022). https://doi.org:10.1016/j.dld.2021.06.011
17 Chalasani, S. H., Syed, J., Ramesh, M., Patil, V. & Pramod Kumar, T. M. Artificial intelligence in the field of pharmacy practice: A literature review. *Exploratory Research in Clinical and Social Pharmacy* **12**, 100346 (2023). https://doi.org:https://doi.org/10.1016/j.rcsop.2023.100346
18 Varghese, C., Harrison, E. M., O'Grady, G. & Topol, E. J. Artificial intelligence in surgery. *Nature Medicine* **30**, 1257-1268 (2024). https://doi.org:10.1038/s41591-024-02970-3
19 Federer, S. J. & Jones, G. G. Artificial intelligence in orthopaedics: A scoping review. *PLoS One* **16**, e0260471 (2021). https://doi.org:10.1371/journal.pone.0260471
20 McGenity, C. *et al.* Artificial intelligence in digital pathology: a systematic review and meta-analysis of diagnostic test accuracy. *npj Digital Medicine* **7**, 114 (2024). https://doi.org:10.1038/s41746-024-01106-8
21 Chen, A. B. *et al.* Artificial Intelligence Applications in Urology: Reporting Standards to Achieve Fluency for Urologists. *Urol Clin North Am* **49**, 65-117 (2022). https://doi.org:10.1016/j.ucl.2021.07.009
22 Bur, A. M., Shew, M. & New, J. Artificial Intelligence for the Otolaryngologist: A State of the Art Review. *Otolaryngology–Head and Neck Surgery* **160**, 603-611 (2019). https://doi.org:https://doi.org/10.1177/0194599819827507
23 Kueper, J. K., Terry, A. L., Zwarenstein, M. & Lizotte, D. J. Artificial Intelligence and Primary Care Research: A Scoping Review. *Ann Fam Med* **18**, 250-258 (2020). https://doi.org:10.1370/afm.2518
24 Park, S. *et al.* Artificial intelligence with kidney disease: A scoping review with bibliometric analysis, PRISMA-ScR. *Medicine (Baltimore)* **100**, e25422 (2021). https://doi.org:10.1097/md.0000000000025422
25 Madrid-García, A., Merino-Barbancho, B., Rodríguez-González, A., Fernández-Gutiérrez, B., Rodríguez-Rodríguez, L. & Menasalvas-Ruiz, E. Understanding the role and adoption of artificial intelligence techniques in rheumatology research: An in-depth review of the literature. *Seminars in Arthritis and Rheumatism* **61**, 152213 (2023). https://doi.org:https://doi.org/10.1016/j.semarthrit.2023.152213
26 Hashimoto, D. A., Witkowski, E., Gao, L., Meireles, O. & Rosman, G. Artificial Intelligence in Anesthesiology: Current Techniques, Clinical

Applications, and Limitations. *Anesthesiology* **132**, 379-394 (2020). https://doi.org:10.1097/ALN.0000000000002960
27 Kotsyfakis, S. *et al.* The application of machine learning to imaging in hematological oncology: A scoping review. *Front Oncol* **12**, 1080988 (2022). https://doi.org:10.3389/fonc.2022.1080988
28 Mörch, C. M. *et al.* Artificial Intelligence and Ethics in Dentistry: A Scoping Review. *Journal of Dental Research* **100**, 1452-1460 (2021). https://doi.org:10.1177/00220345211013808
29 Stafford, I. S., Kellermann, M., Mossotto, E., Beattie, R. M., MacArthur, B. D. & Ennis, S. A systematic review of the applications of artificial intelligence and machine learning in autoimmune diseases. *npj Digital Medicine* **3**, 30 (2020). https://doi.org:10.1038/s41746-020-0229-3
30 Chaudhry, Z. S. & Choudhury, A. Clinical Applications of Artificial Intelligence in Occupational Health: A Systematic Literature Review. *Journal of Occupational and Environmental Medicine* **66** (2024).
31 Sumner, J., Lim, H. W., Chong, L. S., Bundele, A., Mukhopadhyay, A. & Kayambu, G. Artificial intelligence in physical rehabilitation: A systematic review. *Artificial Intelligence in Medicine* **146**, 102693 (2023). https://doi.org:https://doi.org/10.1016/j.artmed.2023.102693
